# Mosquito Repellents: Efficacy Tests of Commercial Skin-Applied Products in China

**DOI:** 10.3390/molecules27175534

**Published:** 2022-08-28

**Authors:** Zhe-Yu Peng, Mu-Zi He, Ling-Yan Zhou, Xin-Yu Wu, Lin-Min Wang, Ni Li, Sheng-Qun Deng

**Affiliations:** The Key Laboratory of Microbiology and Parasitology of Anhui Province, The Key Laboratory of Zoonoses of High Institutions in Anhui, Department of Pathogen Biology, School of Basic Medical Sciences, Anhui Medical University, Hefei 230032, China

**Keywords:** mosquito repellent, efficacy test, N, N-diethyl-m-toluamide, protection time

## Abstract

As a prevention tool for mosquito-borne diseases, mosquito repellents have received substantial attention. To make a convincing recommendation for repellent products to Chinese consumers, we compared the protection time (landing time and probing time) of the 26 best-selling commercial repellents in the Chinese market in a controlled laboratory environment. The data were analyzed by one-way ANOVA. Meanwhile, prices and favorable rates of repellents are also taken into consideration. In our study, N, N-diethyl-m-toluamide (DEET)-based products provided the longest protection time (0.5–3.88 h landing time and/or 1–5.63 h probing time) and lower prices (13.9–21.9 yuan) than other components (ethyl butylacetylaminopropionate (IR3535), picaridin, and botanical. Among the 26 selected products, only 17 repellents showed repellency, and the best repellent was Green Jungle (15% DEET), with a mean (±SD) landing and/or probing time of 3.88 ± 1.65 h and/or 5.63 ± 0.36 h. For botanicals, only ICE King, OMNIbaby, and Ren He showed a little repellency. Autan (20% picaridin) performed best in the picaridin group. Run Ben (7% IR3535) stood out from the IR3535 group. In conclusion, DEET repellent is highly recommended to consumers. The combination of botanicals and synthesized chemicals is a new prospect for eco-friendly repellents.

## 1. Introduction

Due to worldwide trade and global warming, mosquito-borne diseases have witnessed steady growth in the past few years and have caused epidemics around the world. The important mosquito-borne diseases are malaria, West Nile, yellow fever, dengue, chikungunya, and Zika [1]. Owing to the culmination of decades of dedicated efforts, China was certified malaria-free by the World Health Organization (WHO) on 30 June 2021 [2]. However, with the elimination of malaria, dengue became the primary mosquito-borne disease in China. For instance, Guangdong Province had an outbreak of dengue with 45189 cases in 2014 [3]. *Aedes albopictus* is an important vector for the transmission of dengue viruses in mainland China, and females bite aggressively during the day and preferably outdoors [4,5]. It has been reported that southeast China, including Guangdong, Fujian, Zhejiang, Jiangsu, and Jiangxi provinces, presents a higher density of *Ae. albopictus* population than in other areas in China [6]. These provinces also show an increasing drift in dengue fever incidence [7]. A majority of dengue cases were asymptomatic or showed mild symptoms, which made the prevention and control of dengue difficult. Therefore, powerful vector control measures are required to relieve the burden of dengue disease.

To control mosquito-borne diseases, many personal protection measures against mosquito bites have been suggested, such as wearing protection (e.g., hat, light-color cloth including long-sleeved shirts and long pants), mosquito nets, and the use of repellents. Given the unbearable heat in summer when mosquitoes are the most active, skin-applied repellents remained the first choice compared to wearing protection. Repellents are substances that are formulated for use on bare skin to help people avoid mosquito bites [8]. The Environmental Protection Agency (EPA)-registered skin-applied insect repellents that were proven safe and effective generally contained the following active ingredients: catnip oil, oil of citronella, N, N-diethyl-m-toluamide (DEET), ethyl butylacetylaminopropionate (IR3535), picaridin, p-Menthane-3,8-diol (PMD), oil of lemon eucalyptus, and 2-undecanone [9]. Facing these repellent products on the market, Chinese consumers need comprehensive information about which kind of products offer the best repellent efficacy as well as safety at an economical price.

The research was limited to a full range of commercial mosquito repellents, usually of certain repellent ingredients in a specific country. The efficacy test of commercial repellents in China was a blank space. To fill this margin, we compared the repellent effect of 26 commercial skin-applied repellents on the Chinese market under laboratory conditions, and we also collected the price and high praise rate of mosquito repellents to make a more convincing recommendation of the best repellent product for Chinese consumers.

## 2. Results

### 2.1. Selected Repellent Products

**Figure 1:**
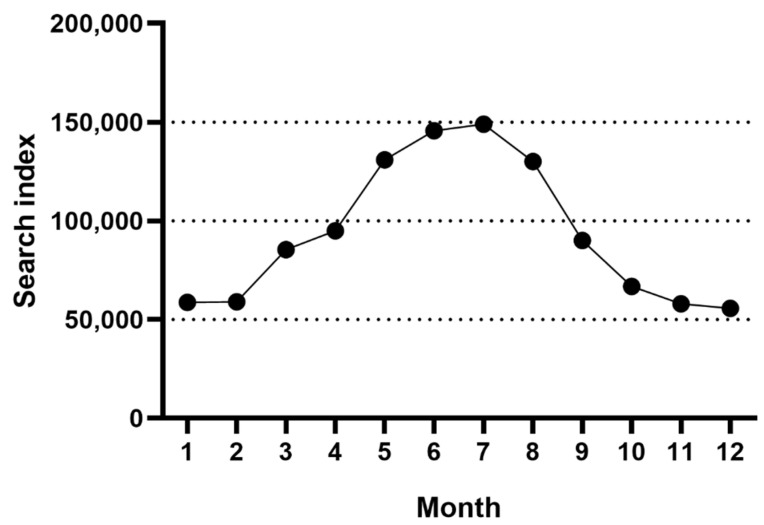
The search index of mosquito repellents in China in 2021.

**Figure 2:**
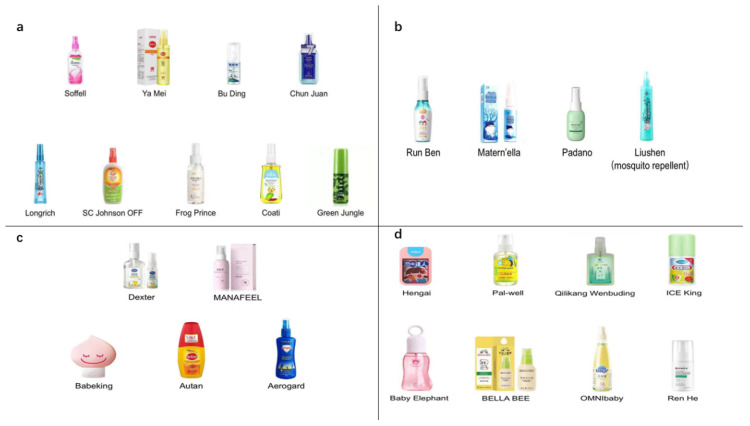
The commercial repellent products selected for testing (26 in total). (**a**) Nine DEET-based repellents; (**b**) four IR3535-based repellents; (**c**) five picaridin-based repellents; (**d**) eight botanical component repellents.

Throughout 2021, the search index of mosquito repellents in China (Baidu index) reached its climax (approximately 150,000) in July (Figure 1). The sales of repellent products in July were collected from the Taobao and Jingdong platforms (Table 1). The most popular repellent in China was Run Ben, with its sales number greater than 261,911 in July. Finally, we purchased the top 26 repellent products with more than 100 sales in July from official flagship online stores. The 26 selected repellents were classified into four kinds regarding the active ingredients DEET, IR3535, picaridin, and botanical components (Figure 2a–d). All the information about the manufacturer, properties, main components, and concentration together with the toxicity of mosquito repellents are available in Table 2.

### 2.2. Repellency Bioassay

Among the four types of repellent products tested, DEET-based products provided the longest protection time (landing time and/or probing time) against *Ae. albopictus* bites. The DEET products (5–15%) provided a landing time ranging from 0.5 to 3.88 h and/or a probing time ranging from 1 to 5.63 h. The picaridin-based repellents (10–20%) provided a shorter landing time (ranging from 0–1.5 h) and/or shorter probing time (ranging from 0.5–2.5 h). The selected botanical products were non-repellent except ICE King, OMNIbaby, and Ren He. Products containing IR3535 (4.5–7%) provided only a landing time (ranging from 0–0.75 h) and/or probing time (ranging from 0.5–1.13 h) (Table 3).

Considering both the mean landing time and mean probing time, only 19 repellents showed repellency, and the top three repellents were Green Jungle (15% DEET), Bu Ding (15% DEET), and Ya Mei (10% DEET). Green Jungle (15% DEET), Bu Ding (15% DEET), and Ya Mei (10% DEET) showed repellent efficacy with a mean (±SD) landing time of 3.88 ± 1.65 h, 2.83 ± 1.75 h, and 2.50 ± 2.05 h and/or a mean (±SD) probing time of 5.63 ± 0.36 h, 4.80 ± 1.25 h, and 4.40 ± 1.14 h. Formulations containing 5% DEET (Longrich, Coati, Frog Prince, and Chun Juan) exhibited a different degree of repellency. Frog Prince presented the mean (±SD) landing time of 1.38 ± 0.48 h. Longrich and Coati had the mean (±SD) probing time of 1.75 ± 0.29 h. The mean landing time and/or probing time of 5%, 7%, 10%, and 15% DEET repellents were compared to determine the impact of concentration on mosquito repellency (y = 0.3545x + 0.1393, R^2^ = 0.8751).

The Autan (20% picaridin) had a similar landing time (mean (±SD), 1.5 ± 0.71 h) to the other two 20% picaridin products (F = 1.695, df = 2, *p* = 0.228), but the probing time (mean (±SD), 2.5 ± 0.5 h) was longer (F = 4.290, df = 2, *p* = 0.045). For the eight selected botanical products, only ICE King, OMNIbaby, and Ren He showed a little repellency against *Ae.* *albopictus*. ICE King had better repellency performance than the other two botanical products with a mean (±SD) landing time of 0.88 ± 0.25 h (F = 4.231, df = 2, *p* = 0.047) and/or a mean probing time of 1.13 ± 0.25 h (F = 10.500, df = 2, *p* = 0.004). Run Ben (7% IR3535) presented a similar landing time (mean (±SD), 0.75 ± 0.35 h) than other IR3535-based products (Matern’ella and Padano) (F = 2.722, df = 2, *p* = 0.133), but had a longer probing time (mean (±SD), 1.13 ± 0.25 h) (F = 10.500, df = 2, *p* = 0.004).

In addition, Liushen (4.5% IR3535), Aerogard (10% picaridin), and five botanical products (Hengai, Pal-well, Qilikang Wenbuding, Baby Elephant, and BELLA BEE) were all non-repellent.

### 2.3. The Price and Favorable Rates of Mosquito Repellents

Given that the favorable rates for Jingdong were all higher than 95%, which lacked comparability, we chose the favorable rates data from Taobao for analysis. However, the average price of each product was based on both Taobao and Jingdong for a fair result. In general, the prices of DEET, IR3535, picaridin, and plant component repellents were 13.9–21.9 yuan, 9.9–26.9 yuan, 28–48 yuan, and 12–39 yuan, respectively (Table 4).

Green Jungle (15% DEET), Bu Ding (15% DEET), and Ya Mei (10% DEET) had mean prices of 16.7 yuan, 21.3 yuan, and 30.3 yuan, respectively, with favorable rates of 95.55%, 95.77%, and 94.85%, respectively. Autan (20% picaridin) had an average price of 49.5 yuan with a mean favorable rate of 98.5%. ICE King (botanical) had a mean price of 24.0 yuan with a favorable rate of 94.45%. Run Ben (7% IR3535) reached an average price of 12.90 yuan with a favorable rate of 93.96% (Table 4).

## 3. Discussion

Avoiding mosquito bites remains an important tool to cut off the transmission routes of mosquito-borne diseases. The application of effective mosquito repellents could avoid mosquito bites at lower costs. The mosquito repellents available are mainly based on DEET, IR3535, picaridin, and botanical components. Over the past decades, DEET has been a gold standard for repellents and is widely used. People tend to spray it on the skin or clothes to avoid mosquito bites. The mechanism of DEET repellent is to provide a vapor barrier that prevents mosquitoes from approaching the skin through the mediation of molecular targets (odorant receptors and ionotropic receptor Ir40a) [10]. Odorant receptors mediate the effect of DEET at a distance, whereas other chemoreceptors mediate repellency upon contact, and Ir40a has been confirmed as a putative DEET chemosensor in Drosophila [11]. The identification of candidate molecular targets for the action of DEET may assist in the design of new potential insect repellents [12]. A concentration of 20% to 50% was recommended for application in malaria and other vector-borne disease areas to provide complete protection against *Aedes*, *Anopheles*, and *Culex* mosquito species for 6 to 13 h [13]. However, 5–15% DEET was more common for daily use, as the popular commercial repellents in the China survey showed (Table 1 and Table 2). Additionally, proven to be an effective repellent under sweating and water leaching, DEET was a perfect match for vigorous physical activity situations [13].

In our study, DEET-based products provided the longest protection time (0.5–3.88 h landing time and/or 1.00 to 5.63 h probing time) at lower prices. The 5% DEET products had a moderate protection effect with the cheapest price (11.90 yuan), and even the 15% DEET, which provided the longest protection time throughout the test, was cheaper than picaridin and other repellents. Previous studies also indicated that the repellent efficacy of 23.8% DEET-based products was remarkable, with an average of 5 h of complete protection time at a single application [14].

Although DEET has an excellent safety profile and remarkable protection against mosquitoes, its toxicity and ecological risk caused by incorrect use or overuse should be carefully discussed [15]. DEET was reported to have neurotoxic and cardiotoxic effects, leading to encephalopathy, respiratory depression, seizure, and coma, as well as profound hypotension [16]. A very high concentration of DEET was detected in the surface water of the Yangtze River of China, which reflected a severe pollution problem caused by the broad use of this repellent [17]. DEET would also dissolve plastics and synthetic rubber products, making it inconvenient for hand skin protection. The exposure of gestating female rats to a DEET and permethrin pesticide combination promoted kidney disease, prostate disease, transgenerational testis disease, and the presence of multiple diseases in the subsequent generation [18].

Picaridin had a shorter protection time against *Ae. albopictus* and higher prices but also possessed many advantages of an ideal repellent. It was odorless, had a better skin-applied feeling, and had less irritation with no damage to plastics or fabrics. However, there were limited picaridin-based commercial repellents on the Chinese market for testing. The 20% concentration of picaridin was considered to protect for up to 12 h against lone star ticks (*Amblyomma americanum*), which was similar to the protective effect of 33% DEET cream and much longer than the protection time of our tested repellents against *Ae. albopictus* [19]. In the DEET-based repellents tested, Green Jungle provided the longest protection time, with a mean (±SD) landing time of 3.88 ± 1.65 h and/or a mean (±SD) probing time of 5.63 ± 0.63 h against *Ae. albopictus* and was highly recommended for daily use.

From our study, full botanical products showed less repellency against mosquito bites than synthesized repellents, but certain components of the products received much attention from botanical repellent research. The main active components of botanical products were menthol and essential oils (i.e., citronella oil and lemon eucalyptus), which created a synergistic repellent effect. Citronella oil, an essential oil widely used in botanical products and a mixture of citronellal, citronellol, and geraniol, exhibited good efficacy under 100% concentration against mosquitoes in a study published in 2015 [20]. However, for some commercial repellents containing 12% or less citronellal, the protection time against mosquito bites was much less than that of DEET and IR3535 [14]. The practical application of citronellal repellents at high concentrations still faces many difficulties due to the poor stability and rapid degradation of citronellal [21]. The oil of lemon eucalyptus was the main active component of Green Jungle, Ren He, and Hengai. Extracted from lemon eucalyptus, PMD was said to be as effective as DEET when used in similar quantities, which indicated a reasonable alternative to DEET and superior to other botanical alternatives [22].

Moreover, the repellent efficacy of a mixture of synthesized chemicals and essential oils might be higher than those of a single formulation. For instance, Green Jungle, consisting of lemon eucalyptus oil and 15% DEET, had a better repellent effect than the single 15% DEET product Bu Ding. Frog Prince, which mainly consists of plant extracts and 5% DEET, performed better regarding the landing time than other pure 5% DEET products, but the probing time of these 5% DEET repellents was very close. Padano, which had a 6% IR3535 blend formulated with botanical components, presented a better repellent effect than Ren He (full botanical) but at a cheaper price. Some new botanical ingredients were reported to be included in the repellents, such as vanillin, liquid paraffin, and salicyluric acid [23]. Formulations supplemented with 5% vanillin had an increase in protection time compared with DEET alone [24]. Furthermore, the combination of citronella oil and vanillin was likely to have a comparable protection time against DEET in *Anopheles* and *Culex* mosquitoes [25]. Some botanical repellents (i.e., lemongrass oil and eugenol) were reported to have a strong effect on small numbers of Anopheles odorant receptor- co-receptor-expressing olfactory receptor neurons at low concentrations, indicating a new prospect for repellent development [26].

In addition, three famous traditional Chinese repellents (cooling ointment, essential balm, and toilet water) were also tested in the laboratory and showed less protective efficacy. In detail, both the cooling ointment and Liushen (gets rid of prickly heat) presented no repellence. The essential balm had a mean (±SD) landing time of 0.67 ± 0.29 h and/or a mean (±SD) probing time of 0.75 ± 0.35 h. Compared with the less effective traditional Chinese repellents, the formulations containing DEET, which have proven to be the best choice of repellents, were neglected by Chinese consumers, with only a medium sales ranking and an 87.99% favorable rate. Therefore, more information regarding the efficacy and safety of repellents is needed to guide consumers about the choice of mosquito repellents in China.

IR3535 was not harmful when ingested, inhaled, or used on skin and did not accumulate in the environment. It was reported that IR3535 could increase nicotinic receptor sensitivity to the neonicotinoid insecticide thiacloprid by activating the orthosteric muscarinic receptor, which proposed a new approach to the integration of repellents [27]. However, IR3535-based repellents did not perform well in the repellency test; only Run Ben (7% IR3535), Padano (6% IR3535), and Matern’ella (6% IR3535) had a repellent effect, and all of them fell far behind the other tested repellents. Apart from that, IR3535 was found to have synergistic effects when co-administered with DEET, and the combination of the two compounds could prolong the protection time as well as reduce toxicity [28]. Another repellent mixture of IR3535 and nonanoic acid had lower liquid vapor pressure than that of the parent compounds and even caused partial mosquito mortality [29].

The suitability and effectiveness of a repellent formulation depend on inherent properties such as vapor pressure, boiling point, and volatility [30]. Previous studies have shown that protection time is inversely proportional to the evaporation rate of the repellents, which is directly proportional to the vapor pressure. The repellents could be ranked in terms of decreasing volatility as DEET > IR3535 > picaridin, which was also theoretically the ranking of their repellent efficacy [31]. Our study showed similar results: DEET surpassed IR3535 and picaridin in the efficacy test, but the repellent efficacy of picaridin shown in the test was slightly better than that of IR3535. The picaridin had a landing time of 0.5–1.5 h and/or probing time of 0.5–2.5 h, whereas IR3535 had only 0.33–0.75 h landing time and/or 0.5–1.13 h. Such a difference might be attributed to the small concentration of commercial IR3535 products with 4.5–7%, whereas picaridin had a wider range of 10–20%.

With various kinds of repellents, consumers must make their own choices based on not only the repellent efficacy and price but also the occasions where repellents are applied. Symptoms of mosquito-borne illnesses in pregnant women usually manifest as pregnancy-specific illnesses, making treatment very difficult [32]. For example, symptoms of vomiting, nausea, and muscle and joint pains were seen both in dengue and pregnancy, which emphasized the use of repellents as preventive agents among pregnant women [33]. Even if all EPA-registered insect repellents are proven safe and effective for pregnant and breastfeeding women, side effects are rare, according to the Centers for Disease Control and Prevention (CDC) official website [34]. For pregnant and breastfeeding women, there were still several tips to be considered regarding the proper application of repellents. For example, do not use repellent directly on skin that is injured or irritated, and avoid the eyes and mouth. Avoid breastfeeding areas and change clothes applied with repellents before breastfeeding infants. Make sure that small children apply repellent under the watch of adults because the ingestion of liquid mosquito repellent in children is very dangerous, leading to acute respiratory distress syndrome [35]. If users want to apply sunscreen together with repellent, please apply sunscreen first and insect repellent second [34]. A previous study of sunscreen and insect repellent interactions indicated that picaridin may decrease percutaneous absorption of both compounds, whereas DEET and sunscreen result in significantly higher absorption of both compounds [36]. As a result, picaridin-based products were more suitable for sunscreen users with lower toxicity than DEET.

Although the data of this study were satisfying, some limitations still deserve to be discussed. The entire experiment was conducted in laboratory conditions with strictly controlled temperature and relative humidity, which could not fully simulate the real environment. A full-field or semi-field experiment was needed for further testing. In addition, to control the bias of the mosquitoes, only laboratory-reared *Ae. albopictus* were used for the efficacy test. Since certain repellents might show different repellent efficacy against different mosquito species, additional species could be included in this experiment. Only the skin of the hands was exposed to mosquitoes, which did not represent the other parts of the body in an outdoor environment. Because the concentrations of IR3535 and picaridin products were limited in the present Chinese market, the linear relationships between concentration and protection time were unable to be analyzed. Perhaps shortly more products of IR3535 and picaridin will emerge on the Chinese market.

DEET has played a leading role in commercial repellent products for many years, but there have been many new experiments on potential repellent compounds that are considered alternatives to DEET. For example, acylpiperidines were confirmed to provide repellency against mosquitoes by disrupting the insect olfactory system in 2020 [37]. Some advanced technologies have already been applied in repellent development, such as 3D pharmacophore modeling and computer-assisted molecular modeling [38]. Biologically synthesized nanoparticles through plant extracts, essential oils, and conventional pesticides such as pyrethroids are eco-friendly with better target specificity [39]. With a microencapsulation technique, pyrethroids and repellents were mixed for better washing durability, and the encapsulation of botanicals reduced their toxicity to the environment [21,40]. To create a friendly environment, plant-based bioinsecticides, much safer and less toxic, are designed to replace synthetic products [19]. Ingredients such as nicotine, azadirachtin, rotenone, pyrethrin, and other plant secondary metabolites were combined in the repellent products.

## 4. Materials and Methods

### 4.1. Repellent Product Selection

First, we inquired about the search index of mosquito repellents in China throughout 2021 (Baidu index) to describe the repellent viewings by month. Next, the sales of repellent products in the highest-viewings month were collected from the commonly used e-commerce platforms Taobao and Jingdong. Last, the repellent products with high sales (>100) were included in the experiment (Table 1).

### 4.2. Test Mosquitoes

The Foshan (Guangdong, China) strain of *Ae. albopictus*, collected from Foshan City in 1983, was used in this study. This mosquito was kept in the laboratory without insecticide exposure. All mosquitoes, including larval and adult mosquitoes, were maintained at 28 ± 1 °C and 80 ± 5% relative humidity under a photoperiod cycle of 14:10 h (light/dark) in the insectary.

### 4.3. Human Volunteers

Six adult volunteers (three males and three females aged 18 to 22 years) participated in the experiments. They were non-tobacco users and had no history of dermatosis or allergic reactions to arthropod bites or repellents. Given that various factors may alter a person’s attractiveness to mosquitoes, which may, in turn, affect the results of mosquito repellent tests, all the volunteers were required not to apply any aromatics, skincare, or other repellent products on the skin of the hands 12 h before and during the experiment. The experiment was approved by the Medical Ethics Committee of Anhui Medical University (approval code: 2022H002).

### 4.4. Efficacy Testing of Mosquito Repellents

**Figure 3:**
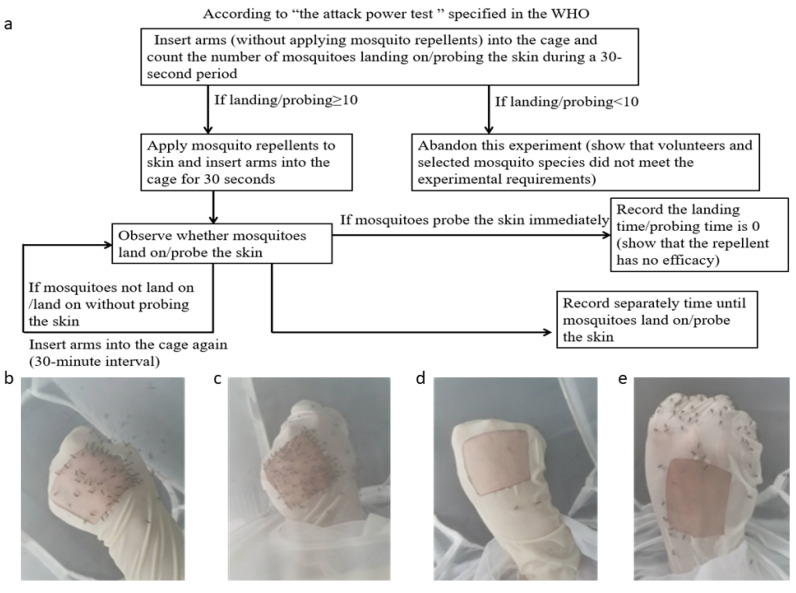
A mosquito attack power testing: (**a**) pre-experiment design; (**b**,**c**) more than 10 mosquitoes landed/probed; (**d**,**e**) less than 10 mosquitoes landed/probed. Only if the biting rate was ≥10 landings and/or probings in 30 s, the mosquitoes and volunteers were qualified for further testing.

The efficacy comparison of 26 commercial skin-applied repellents against *Ae. albopictus* females were conducted using the guidelines for the efficacy testing of mosquito repellents for human skin by the WHO [41]. A total of 305 7-day-old starved female *Ae. albopictus* were placed in a net cage (40 × 30 × 30 cm) before the experiment. The tests were carried out between 15:00 and 20:00 h in accordance with the mosquito biting habits of *Ae. albopictus* in the 24-h cycle. Volunteers wore long-sleeve white coats to cover their arms, sterilized their hands and wrists with unscented soap, rinsed with water, rinsed with a solution of 70% ethanol or isopropyl alcohol in water, and put on gloves after drying hands with towels. Specific rubber gloves were designed to expose only a 4 × 4 cm area on the back of the hand skin (Figure 3a–e), and the gloves were replaced in each test in case of the repellent residue effect.

**Figure 4:**
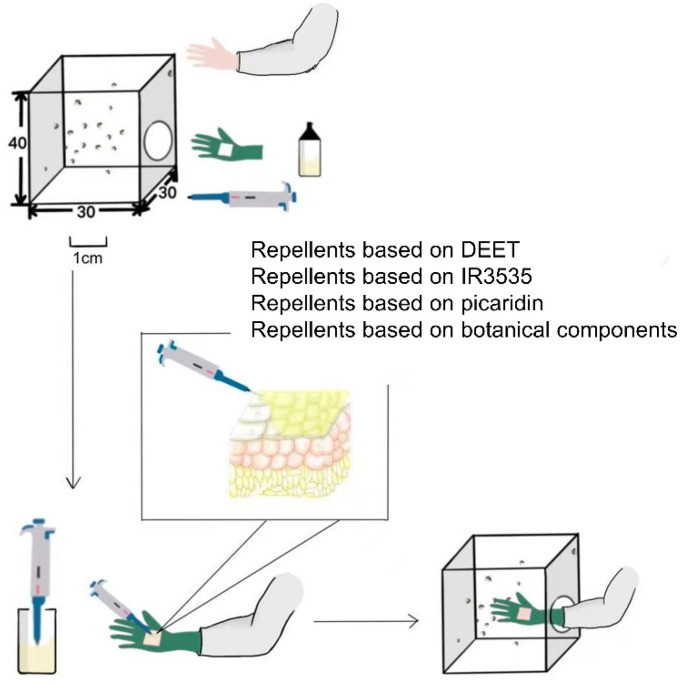
A brief process of efficacy testing of mosquito repellents for human skin in the laboratory.

The first step was to insert the untreated hand into the cage and to count the number of mosquitoes that landed on and/or probed the skin in 30 s. During testing, the volunteer should avoid movement of the hand. Only if the biting rate was ≥10 landings and/or probings in 30 s, the mosquitoes and volunteers were qualified for further testing (Figure 3). The hand was then treated with 50 µL of a repellent product on the exposed 4 × 4 cm skin area, allowed to dry for 1 min, and then inserted into the cage for 30 s (Figure 4). The insertion was repeated at 30-min intervals until the hands received one or more landings and/or probings. Landing and/or probing behavior implies the ending point of the test. A repellent provides efficacy by a reduction in the biting and/or landing activity of mosquitoes. However, landing is not always related to probing, and separate recordings of each behavior are required [41]. The landing and probing times were estimated from the application of the tested products to the mosquitoes’ first biting or landing. Replicate tests repeated this process using different repellents over several weeks. Each repellent had five or six replicate tests conducted per volunteer, allowing the number of results sufficient for statistical analysis. Moreover, volunteers rotated their positions during every test to reduce the potential factors of disturbing the repellent’s efficacy such as positions, catching ability, and the status of mosquitoes. The mosquitoes were allowed a 30-min recovery time after 10 successive tests.

### 4.5. Prices and Favorable Rate of Repellents

We collected the numbers of consumers who gave a favorable reception to a certain repellent by browsing product webpages of Taobao and Jingdong e-commerce platforms and calculated the average favorable rate of repellents. Meanwhile, the price tags of repellents were also recorded to obtain an average.

### 4.6. Statistical Analysis

The data from the experiments were collected and analyzed to compare the repellent effect of different commercial repellent products against *Ae. albopictus* in a laboratory bioassay. The protection efficacy was presented as the mean ± SD of the landing time and probing time. A generalized linear model in GraphPad Prism (8th edition) was used in the regression analysis. Given that the insertion interval was 0.5 h, a product was deemed non-repellent if the landing time was less than 0.5 h. The concentration of the main repellent components was included as an independent variable, whereas the complete protection time was included as the dependent variable in a linear relationship. The favorable rate of a repellent was calculated by the following formula:

$$\mathrm{Favorable}\;\mathrm{rate}=\frac{\sum_{i=1}^{n}\frac{N_{i}}{T_{i}}}{n}\times 100\%$$

where *N* is the number of consumers who gave a favorable reception to a repellent at one store, *T* is the total number of consumers buying this repellent in the store, and *n* is the number of stores selling the repellent on the e-commerce platform. The average price of repellents was also analyzed similarly.

Furthermore, a one-way ANOVA was applied to compare the landing time and probing time of different mosquito repellent products. All analyses were performed with IBM SPSS (v. 20.0), and significance was defined by *p* < 0.05.

## 5. Conclusions

The results from this study have demonstrated that DEET is still a gold standard of repellents with cheaper prices, proving more effective than the other commercial repellents marketed in China for protecting against mosquito bites. In general, Green Jungle (15% DEET) is most recommended for its powerful repellent efficacy and cheap price. Meanwhile, a majority of the botanical products tested are non-repellent and much more expensive than other products. However, the combination of botanical and synthesized chemicals presents a better effect than the single compound, indicating a new prospect for repellents. Given the features of different main components, Autan (20% picaridin) could be the substitute for DEET for those who cared more about skin feel. Run Ben (7% IR3535) is specifically suitable for younger children with safety concerns. However, further studies are required in the future with more kinds of repellents on the Chinese market, and the best choice of repellent may be replaced by more environmentally friendly products one day.

## Figures and Tables

**Table 1:** Sales of various mosquito repellent products on e-commerce platforms Taobao and Jingdong in July.

Mosquito Repellent Products	Sales	Ranking
Run Ben (7% IR3535)	>261,911	1
Babeking (15% picaridin)	>43,716	2
Liushen (mosquito repellent) (4.5% IR3535)	>31,167	3
Matern’ella (6% IR3535)	>25,280	4
Soffell (12% DEET)	>11,415	5
Longrich (5% DEET)	>6650	6
Frog Prince (5% DEET)	>5534	7
Ya Mei (10% DEET)	>4848	8
Chun Juan (5% DEET)	>4662	9
Dexter (20% picaridin)	>4000	10
SC Johnson OFF (7% DEET)	>2392	11
Padano (6% IR3535)	>2000	12
Autan (20% picaridin)	>2000	13
Green Jungle (15% DEET)	>1047	14
Bu Ding (15% DEET)	>1026	15
ICE King (botanical)	>1000	16
Qilikang Wenbuding (botanical)	>695	17
Pal-well (botanical)	>420	18
Coati (5% DEET)	>377	19
Aerogard (10% picaridin)	>324	20
BELLA BEE (botanical)	>235	21
Baby Elephant (botanical)	>229	22
Hengai (botanical)	>200	23
MANAFEEL (20% picaridin)	>200	24
OMNIbaby (botanical)	>100	25
Ren He (botanical)	>100	26

**Table 2:** Manufacturer, properties, main components, and toxicity of selected mosquito repellents.

Mosquito Repellents	Main Components	Toxicity
Longrich	5% DEET, ethanol, benzophenone-4	Slightly toxic
Coati	5% DEET	Slightly toxic
Frog Prince	5% DEET, wild chrysanthemum extract, honeysuckle extract	Slightly toxic
Chun Juan	5% DEET	Slightly toxic
SC Johnson OFF	7% DEET	Slightly toxic
Ya Mei	10% DEET	Slightly toxic
Soffell	12% DEET	Slightly toxic
Bu Ding	15% DEET	Slightly toxic
Green Jungle	15% DEET, glycerol, menthol	Slightly toxic
Liushen	4.5% IR3535, alcohol, menthol, artificial bezoar, artificial musk	Slightly toxic
Matern’ella	6% IR3535, chrysanthellum indicu flower water, rosmarinus officinalis leaf extract	Slightly toxic
Padano	6% IR3535, menthol, alcohol, chrysanthemum extract, leaf extract	Slightly toxic
Run Ben	7% IR3535, honeysuckle, mugwort	Slightly toxic
Aerogard	10% picaridin, alcohol denat, water	Slightly toxic
Babeking	15% picaridin, ethanol, deionized water	Slightly toxic
Dexter	20% picaridin, alcohol	Slightly toxic
MANAFEEL	20% picaridin	Slightly toxic
Autan	20% picaridin	
ICE King	Ethanol, wild chrysanthemum, mint leaf and wormwood leaf extract	Non-toxic
OMNIbaby	Water, ethanol, citronella oil, menthol, eucalyptus oil	Non-toxic
Hengai	Ethanol, citronella extract, lemon eucalyptus leaf extract	Non-toxic
Pal-well	Lemon citronella leaf oil, borneol, menthol	Non-toxic
Qilikang Wenbuding	Honeysuckle, menthol, camphor, citronella, mozzie buster	Non-toxic
Ren He	Ethanol, citronella oil, lemon oil, lemon eucalyptus oil	Non-toxic
Baby Elephant	Ethanol, chrysanthemum extract	Non-toxic
BELLA BEE	Ethanol, wild chrysanthemum extract, citronella leaf oil, menthol	Non-toxic

**Table 3:** Protection time of commercial mosquito repellents against *Ae. albopictus* bites ^1^.

Mosquito Repellent Products	Landing Time (Hours)		Probing Time (Hours)		Number of Subjects
	Mean ± SD	Range	Mean ± SD	Range	
Longrich (5% DEET)	1 ± 0.71	0–1.5	1.75 ± 0.29	1.5–2	5
Coati (5% DEET)	1.33 ± 0.76	0.5–2	1.75 ± 0.29	1.5–2	5
Frog Prince (5% DEET)	1.38 ± 0.48	1.5–2	1.63 ± 0.25	1.5–2	5
Chun Juan (5% DEET)	1.13 ± 0.25	1–1.5	1.38 ± 0.25	1–1.5	5
SC Johnson OFF (7% DEET)	1.5 ± 0.71	0.5–2	1.75 ± 0.29	1.5–2	5
Ya Mei (10% DEET)	2.5 ± 2.05	1.0–5.5	4.4 ± 1.14	3.0–5.5	6
Soffell (12% DEET)	0.5 ± 0	0.5	1 ± 0	1	5
Bu Ding (15% DEET)	2.83 ± 1.75	1.5–4.5	4.8 ± 1.25	4–7	6
Green Jungle (15% DEET)	3.88 ± 1.65	2–5.5	5.63 ± 0.63	5–6.5	5
Liushen (4.5% IR3535)	0	0	0.5 ± 0	0.5	6
Matern’ella (6% IR3535)	0.63 ± 0.25	0.5–1	0.63 ± 0.25	0.5–1	6
Padano (6% IR3535)	0.75 ± 0.29	0.5–1.5	0.75 ± 0.29	0.5–1.5	6
Run Ben (7% IR3535)	0.75 ± 0.35	0–1	1.13 ± 0.25	1–1.5	6
Babeking (15% picaridin)	1 ± 0.5	0.5–1.5	1 ± 0.5	0.5–1.5	5
Dexter (20% picaridin)	1.5 ± 0.71	1–2	2 ± 0	2	5
MANAFEEL (20% picaridin)	1 ± 0.71	0–1.5	2 ± 0	2	5
Aerogard (10% picaridin)	0	0	0.5 ± 0	0.5	5
Autan (20% picaridin)	1.5 ± 0.32	1–2	2.5 ± 0.5	2–3	5
ICE King (botanical)	0.88 ± 0.25	0–1.5	1.13 ± 0.25	1–1.5	5
OMNIbaby (botanical)	0.5 ± 0	0.5	1 ± 0.84	0.5–2	6
Hengai (botanical)	0	0	0.63 ± 0.25	0.5–1	5
Pal-well (botanical)	0	0	0.5 ± 0	0.5	5
Qilikang Wenbuding (botanical)	0	0	0.5 ± 0	0.5	5
Ren He (botanical)	0.63 ± 0.25	0.5–1	0.63 ± 0.25	0.5–1	6
Baby Elephant (botanical)	0	0	0.5 ± 0	0.5	6
BELLA BEE (botanical)	0	0	0.5 ± 0	0.5	5

^1^ Plus–minus values are the means ± SDs of the times to the first bite in the tests of all 26 products. The complete protection time for each product is estimated from the application of repellents to the first mosquito landing and/or probing.

**Table 4:** Price and favorable rate of mosquito repellents.

Mosquito Repellents (Content)	Average Price (Yuan)	Taobao Favorable Rate of Store (%)	Average Price (Yuan)	Jingdong Favorable Rate of Store (%)
Dexter (20 mL)	39.0	97.50	29.0	97.00
Run Ben (50 mL)	11.9	89.91	13.9	98.00
Padano (45 mL)	17.9	95.02	18.9	98.00
Liushen (mosquito repellent) (180 mL)	26.9	90.11	19.8	99.00
Matern’ella (33 mL)	9.9	90.61	21.9	99.00
Ya Mei (150 mL)	30.0	90.61	30.6	99.00
Bu Ding (50 mL)	19.8	95.54	22.8	96.00
OMNIbaby (200 mL)	16.0	96.05	28.5	100.00
Longrich (60 mL)	11.9	89.16	11.9	99.00
Green Jungle (30 mL)	22.0	94.00	11.4	97.09
Coati (100 mL)	14.9	90.00	12.9	98.00
Chun Juan (95 mL)	9.9	91.40	11.9	99.00
SC Johnson OFF (59 mL)	12.9	84.85	15.9	98.00
Frog Prince (100 mL)	9.9	81.41	8.9	98.00
BELLA BEE (30 mL)	9.9	99.52	10.9	97.00
Baby Elephant (95 mL)	39.0	92.36	39.0	97.00
Ren He (50 mL)	39.0	93.74	29.9	97.00
ICE King (50 mL)	24.0	88.89	24.0	100.00
Qilikang Wenbuding (100 mL)	12.0	92.90	12.0	95.00
Pal-well (125 mL)	38.0	94.05	38.0	96.00
Soffell (80 mL)	36.0	95.32	36.0	98.00
Hengai (40 mL)	36.0	97.91	36.0	100.00
MANAFEEL (60 mL)	48.0	95.60	48.0	100.00
Autan (100 mL)	50.0	99.00	49.0	98.00
Aerogard (175 mL)	29.0	97.93	30.0	98.00
Babeking (20 mL)	39.9	95.97	39.9	97.00

## Data Availability

The datasets generated and/or analyzed during the current study are available from the corresponding author on reasonable request.
